# Metagenomic next-generation sequencing of matched donor–recipient pairs reveals pathogen transmission dynamics and informs antimicrobial therapy in liver transplantation: a retrospective cohort study

**DOI:** 10.3389/fcimb.2026.1919428

**Published:** 2026-10-19

**Authors:** Shuai Feng, Jinquan Liu, Xiaojing Zhang, Lianghao Zhang, Shengbao Zhao, Nannan Jiang, Jinzhen Cai, Yafeng Zheng, Wei Gai, Chuanshen Xu

**Affiliations:** 1 Organ Transplantation Center, The Affiliated Hospital of Qingdao University, Qingdao, Shandong, China; 2 WillingMed Technology (Beijing) Co., Ltd, Beijing, China; 3 Department of Orthopaedics, The Affiliated Hospital of Qingdao University, Qingdao, Shandong, China

**Keywords:** antimicrobial stewardship, donor-derived infection, liver transplantation, metagenomic next-generation sequencing, post-transplant infection

## Abstract

**Background:**

Post-transplant infections remain a leading cause of morbidity and mortality after liver transplantation (LT). Conventional microbiological culture has well-recognized limitations in sensitivity and pathogen spectrum coverage. Metagenomic next-generation sequencing (mNGS) has emerged as a promising diagnostic platform. However, its systematic evaluation across matched donor–recipient pairs, including characterization of possible donor-to-recipient pathogen transmission and its impact on antimicrobial stewardship, remains limited.

**Methods:**

We retrospectively enrolled 45 consecutive LT donor–recipient pairs. Multiple sample types were collected from donors (*n* = 124) and recipients (*n* = 97) and subjected to both mNGS and conventional culture. Recipients were stratified into infection (*n* = 29) and non-infection (*n* = 16) groups. Pathogen detection rates, diagnostic performance metrics, donor-to-recipient transmission events, and mNGS-informed antimicrobial modifications were systematically analyzed.

**Results:**

Post-transplant infection occurred in 64.4% of recipients, predominantly involving the pulmonary compartment (89.7%). mNGS achieved significantly higher overall positivity rates than culture in both donors (95.6% *vs*. 80.0%) and recipients (64.4% *vs*. 22.2%), with superior detection of fungi and exclusive identification of all viral pathogens. The pathogen-level concordance between methods was predominantly partial (75%–78%). At the patient level, mNGS demonstrated a sensitivity of 69.0% *versus* 31.0% for culture, while culture maintained higher specificity (93.8% *vs*. 43.8%). A diverse pathogen landscape was identified, including *Pneumocystis jirovecii*, rare molds, cytomegalovirus (CMV), and Epstein–Barr virus (EBV)—all exclusively detected by mNGS. A total of 11 possible donor-to-recipient transmission events involving six pathogens were identified across 11 pairs, and 54.5% of affected recipients developed clinical infections, with *Klebsiella pneumoniae* accounting for the majority of transmission events. mNGS results informed antimicrobial treatment modifications in 69.0% of infected recipients. In the univariate analysis, liver cancer showed an exploratory association with lower post-transplant infection risk (OR = 0.10, 95%CI = 0.02–0.46, *p* = 0.003).

**Conclusions:**

This retrospective matched-pair study suggests that mNGS substantially expands pathogen detection beyond conventional culture, enabling the identification of clinically relevant possible donor-derived pathogen transmission and informing antimicrobial treatment modifications in the majority of infected recipients. These findings support mNGS as an adjunctive peri-transplant diagnostic tool pending prospective multicenter validation.

## Introduction

Liver transplantation (LT) is the definitive treatment for end-stage liver disease and early hepatocellular carcinoma, with over 30,000 procedures performed annually worldwide (Meng et al., 2024). Despite advances in surgical technique, immunosuppressive management, and postoperative care, infectious complications remain a principal driver of early post-transplant morbidity and mortality. Recent epidemiological data indicate that more than 60%–80% of solid organ transplant (SOT) recipients develop at least one clinically significant infection within the first year following transplantation (Peng et al., 2024; Van Den Daele et al., 2024). Among LT recipients specifically, early postoperative infection rates in contemporary cohorts range from 44% to 71%, with pulmonary, abdominal, and bloodstream infections being the most prevalent manifestations (Zhao et al., 2022; Zhuang et al., 2024). A recent 13-year single-center experience similarly reported that 78.2% of surviving recipients developed at least one post-transplant infection within the first year, predominantly bacterial and Gram-negative in origin (Kurt et al., 2026). The net state of immunosuppression imposed by induction and maintenance regimens profoundly reshapes the host’s susceptibility to a broad spectrum of bacterial, fungal, and viral pathogens, including opportunistic organisms rarely encountered in immunocompetent individuals (Mezochow et al., 2024).

Timely and accurate pathogen identification is the cornerstone of effective infection management in this population. However, conventional microbiological culture, which is the long-standing diagnostic standard, is limited by slow turnaround times, poor sensitivity for fastidious or low-abundance organisms, inability to detect viruses, and significant susceptibility to prior antibiotic exposure (Yang et al., 2024). These constraints are particularly consequential in the transplant setting, where empirical broad-spectrum antimicrobial therapy is common and delayed targeted treatment is associated with worse clinical outcomes. A recent systematic evaluation of post-LT infections reported culture sensitivities for bacteremia as low as 3%–22%, underscoring the diagnostic gap in routine clinical practice (Huang et al., 2022; Zhao et al., 2022).

Metagenomic next-generation sequencing (mNGS) has emerged as a powerful culture-independent diagnostic platform that simultaneously detects bacteria, fungi, viruses, and parasites directly from clinical specimens without *a priori* hypothesis regarding the causative pathogen (Miller et al., 2019). By sequencing all nucleic acids in a sample and aligning reads against comprehensive microbial reference databases, mNGS can identify organisms across the full taxonomic spectrum, including uncommon or novel pathogens. Accumulating evidence in immunocompromised populations has consistently demonstrated the superior sensitivity of mNGS over conventional culture (58%–75% *vs*. 17%–22%), with the greatest diagnostic advantage observed in the detection of fungi and viruses such as *Pneumocystis jirovecii*, cytomegalovirus (CMV), and Epstein–Barr virus (EBV) (Wang et al., 2025; Zheng et al., 2025). In the specific context of LT, early studies have demonstrated higher pathogen positivity rates with mNGS and its potential to inform antimicrobial treatment modifications with favorable clinical outcomes (Huang et al., 2022; Zhuang et al., 2024).

A critical yet underappreciated dimension of infection risk in LT is the contribution of donor-derived pathogens. During the transplant procedure, microbial organisms harbored in the donor’s respiratory tract, bloodstream, or organ preservation solution may be directly transmitted to the recipient through the allograft or surrounding fluids (Nam et al., 2018). Donor-derived infections (DDIs) have been reported at incidence rates of approximately 16%–19% in kidney and liver transplant cohorts and are associated with increased infection events, elevated bilirubin levels, graft loss, and prolonged hospitalization (Zhou et al., 2026). Carbapenem-resistant *Klebsiella pneumoniae* and other multidrug-resistant Gram-negative organisms have been documented as causative agents of fatal DDIs, particularly when pre-transplant donor screening fails to identify high-risk colonization (Giani et al., 2014; Varotti et al., 2016). Despite the recognized clinical significance of DDIs, systematic multi-compartment pathogen profiling of donors using high-sensitivity methods such as mNGS remains uncommon, and the true spectrum of donor-to-recipient pathogen transmission has not been comprehensively characterized.

Furthermore, existing studies on mNGS in LT have predominantly focused on the recipient alone, or have evaluated mNGS in a single sample type. Comparative analyses across simultaneously collected, matched donor–recipient pairs covering multiple anatomical compartments are lacking. Such data are essential for understanding the bidirectional microbiological relationship between donor and recipient, establishing the diagnostic value of mNGS relative to culture across different sample matrices and evaluating the real-world clinical impact of mNGS-informed antimicrobial decision-making.

In this retrospective cohort study of 45 matched LT donor–recipient pairs, we systematically characterized the pathogen spectrum identified by mNGS and conventional culture across multiple sample types in both donors and recipients, quantified the donor-to-recipient pathogen transmission events, evaluated the diagnostic performance of both methods for the detection of post-transplant infection, and assessed the impact of mNGS-informed findings on antimicrobial modifications. These findings provide preliminary evidence supporting the integration of mNGS into peri-transplant microbiological surveillance and antimicrobial stewardship and underscore the need for prospective, multicenter validation.

## Materials and methods

### Study design and participants

This is a retrospective cohort study conducted at the Department of Liver Transplantation of the Affiliated Hospital of Qingdao University between May 2024 and January 2025. The study enrolled LT donors and their corresponding recipients during the study period. Eligible donors had at least one biological sample submitted for mNGS testing during ICU admission prior to organ procurement. Recipients were eligible if they had available clinical follow-up data for at least 30 days post-transplantation. Recipients with incomplete medical records or those lost to follow-up were excluded.

Among the 89 enrolled donors, 45 had corresponding recipients with complete paired clinical and microbiological data. This matched donor–recipient subset formed the basis for all paired analyses, including pathogen transmission, diagnostic performance evaluation, and treatment adjustment assessment.

### Sample collection

For all enrolled donors, samples were collected for pathogen testing within 24 h prior to organ procurement. Three sample types were obtained where clinically feasible: 1) sputum or endotracheal aspirate; 2) EDTA anti-coagulated peripheral blood; and 3) organ preservation solution (OPS), collected aseptically at the time of organ perfusion during procurement surgery. All samples were processed within 24 h of collection.

For enrolled recipients, samples were collected within 24 h postoperatively. Sample types collected included: 1) sputum or bronchoalveolar lavage fluid (BALF); 2) EDTA peripheral blood; 3) ascites (when abdominal infection was suspected and peritoneal fluid was accessible); and 4) OPS.

### mNGS testing

All samples were submitted to WillingMed Technology (Beijing) Co., Ltd. (Beijing, China) for mNGS analysis. DNA extraction and library preparation were performed according to the manufacturer’s standardized protocols (Liu et al., 2022; Jiang et al., 2024). Sequencing was performed on the MGISEQ-200 platform (MGI Tech, Shenzhen, China). After removal of human host sequences using the hg38 reference genome, remaining reads were aligned against a comprehensive microbial reference database (NCBI GenBank/RefSeq/nt) encompassing bacteria, fungi, viruses, and parasites using the proprietary bioinformatics pipeline of WillingMed Laboratory.

The microbial abundance of each pathogen was quantified using RPTM (reads per 10 million), defined as the number of microbe-specific reads detected per 10 million raw sequencing reads. A threshold of RPTM ≥ 3 was set for viral positivity. For bacteria and fungi, the positive cutoff was defined as RPTM ≥ 20. For special pathogens, including *Cryptococcus* and *Mycobacterium tuberculosis*, an RPTM value ≥1 was considered positive.

The clinical significance of each detected microorganism was interpreted by two experienced clinical experts, integrating the mNGS results with clinical presentation, laboratory parameters, imaging findings, conventional microbiological results, and the response to therapy. Organisms detected in the absence of compatible clinical evidence of infection were interpreted as colonization, contamination, or clinically insignificant microbial signals rather than as confirmed pathogens.

### Conventional microbiological testing

Conventional culture was performed in parallel with mNGS for all collected samples. Culture incubation followed standard laboratory protocols. Organism identification was performed using matrix-assisted laser desorption/ionization time-of-flight mass spectrometry (MALDI-TOF MS). Antimicrobial susceptibility testing was conducted using broth microdilution or disc diffusion in accordance with the Clinical and Laboratory Standards Institute (CLSI) guidelines.

### Definitions

Donor-to-recipient pathogen transmission was defined as possible donor-derived transmission when the same microbial species was detected in both the donor and matched recipient within the peri-transplant period. These events represent species-level concordance rather than confirmed transmission.

mNGS-informed treatment modification was defined as any modification to the antimicrobial regimen (e.g., escalation, de-escalation, addition of antifungal/antiviral agents, or change in combination therapy) made within 72 h of receipt of an mNGS report, where the treating physician documented that the adjustment was based on the mNGS result. A single patient may have received multiple adjustment types.

Post-transplant infection was defined as a clinically diagnosed infection occurring after transplantation. The final clinical diagnosis was adjudicated by the transplant surgeons and infectious disease specialists based on compatible clinical manifestations, radiological/endoscopic or intraoperative findings, inflammatory markers, conventional microbiological evidence, and documented response to antimicrobial therapy. This clinically adjudicated diagnosis served as the reference standard for the diagnostic performance analysis. Because mNGS was performed as part of routine clinical care in this retrospective cohort, the clinicians establishing the final diagnoses were not blinded to the mNGS results. For organisms with a well-recognized propensity for colonization (e.g., *Staphylococcus aureus*, *Corynebacterium* spp., or *Candida* spp. in respiratory specimens), detection was interpreted as colonization rather than infection unless accompanied by specimen quality criteria consistent with a true lower respiratory tract sample and clinical/radiological/laboratory evidence meeting the pre-specified infection definition.

### Outcome measures

The primary outcome was the diagnostic performance of mNGS *versus* conventional culture for post-transplant infection detection, expressed as sensitivity, specificity, positive predictive value (PPV), and negative predictive value (NPV) with 95% confidence intervals (CIs) calculated with the Wilson score method using the clinically adjudicated post-transplant infection diagnosis (see “*Definitions*”) as the reference standard. Secondary outcomes included: 1) overall and pathogen category-specific mNGS and culture positivity rates in donors and recipients; 2) possible donor-to-recipient pathogen transmission events; 3) concordance between mNGS and culture at the sample and pathogen levels; 4) the proportion and type of antimicrobial treatment adjustments informed by mNGS *versus* those informed by culture; and 5) risk factors for post-transplant infection.

### Statistical analysis

Categorical variables are presented as frequencies and percentages and were compared using the chi-squared test or Fisher’s exact test, as appropriate. Continuous variables are presented as the median with interquartile range (IQR) and were compared using the Mann–Whitney *U* test (two groups) given non-normal distribution confirmed by the Shapiro–Wilk test. Because multiple sample types were frequently collected from the same donor or recipient, sample type-stratified comparisons—in which each individual contributed at most one sample per type—were analyzed using McNemar’s test for paired binary data. For comparisons pooling samples across multiple types from the same individual, generalized estimating equations (GEE) with an exchangeable working correlation structure and robust standard errors, clustering on subject identity, were used as a sensitivity analysis to account for within-subject correlation. Sample-level concordance between mNGS and culture positivity was assessed using Cohen’s kappa coefficient (*κ*), with *κ* values interpreted as: <0.20, slight; 0.21–0.40, fair; 0.41–0.60, moderate; 0.61–0.80, substantial; and >0.80, almost perfect agreement. The pathogen-level concordance among dually positive samples was quantified using the Jaccard similarity index (*J*), calculated as the number of shared pathogen species divided by the total number of unique species detected by either method: *J* = 1.00 indicates complete concordance, while *J* = 0 indicates complete discordance. Partial concordance was defined as 0 < *J* < 1.00. Risk factors for post-transplant infection were assessed using univariate logistic regression. Variables with *p* < 0.10 in the univariate analysis were candidates for multivariable analysis. However, given the limited sample size (*n* = 45 recipients), multivariable modeling was not performed to avoid overfitting. The results are presented as odds ratios (ORs) with 95%CIs. All statistical analyses were performed using R version 4.3.1 (R Foundation for Statistical Computing, Vienna, Austria) and SPSS version 26.0 (IBM Corp., Armonk, NY, USA). A two-sided *p*-value <0.05 was considered statistically significant. For each analysis, the unit of analysis is stated explicitly: the individual recipient was the unit for patient-level diagnostic performance and risk factor analyses, while the individual sample was the unit for sample-level detection and concordance analyses. Descriptive sample-level concordance indices (Cohen’s *κ* and the Jaccard similarity index) treated each sample as an independent observation and were not adjusted for within-subject clustering. These indices should therefore be interpreted as descriptive.

## Results

### Clinical characteristics and infection profile of liver transplant recipients

Of the 45 liver transplant recipients enrolled, 29 (64.4%) developed post-transplant infections (infection group) and 16 (35.6%) remained infection-free (non-infection group). The baseline demographic characteristics, including the age (median of 55.0 years in both groups) and sex distribution (72.4% *vs*. 81.2% male patients), were comparable between groups (both *p* > 0.05). The prevalence of underlying comorbidities—including acute liver failure, hypertension, diabetes mellitus, and immunocompromised status—did not differ significantly. Notably, liver cancer was significantly less common in the infection group (31.0% *vs*. 81.2%, *p* = 0.002). No significant differences were observed in the perioperative variables, including immunosuppressive induction, vasopressor use, or early graft dysfunction. Recipients who developed infection experienced significantly longer ICU stays (median = 91.0 *vs*. 53.9 h, *p* = 0.028), total hospitalization (44.0 *vs*. 23.0 days, *p* = 0.009), and postoperative stay (29.0 *vs*. 21.0 days, *p* = 0.016), reflecting a substantially greater clinical burden in infected recipients (**Table 1**).

**Table 1:** Baseline characteristics of liver transplant recipients.

Variable	Overall (*N* = 45)	Infection (*n* = 29)	Non-infection (*n* = 16)	*P*-value
Age (years), median (IQR)	55.0 (51.0–58.0)	55.0 (50.0–60.0)	55.0 (51.0–57.2)	0.831
Male sex, *n* (%)	34 (75.6%)	21 (72.4%)	13 (81.2%)	0.72
Underlying disease				
Liver cancer, *n* (%)	22 (48.9%)	9 (31.0%)	13 (81.2%)	0.002
Acute liver failure, *n* (%)	21 (46.7%)	16 (55.2%)	5 (31.2%)	0.212
Hypertension, *n* (%)	8 (17.8%)	6 (20.7%)	2 (12.5%)	0.691
Diabetes, *n* (%)	10 (22.2%)	7 (24.1%)	3 (18.8%)	>0.999
Immunocompromised, *n* (%)	13 (28.9%)	8 (27.6%)	5 (31.2%)	>0.999
Re-transplantation, *n* (%)	2 (4.4%)	2 (6.9%)	0 (0.0%)	0.531
Prior surgery history, *n* (%)	34 (75.6%)	21 (72.4%)	13 (81.2%)	0.72
Perioperative variables				
Induction (rATG), *n* (%)	20 (44.4%)	14 (48.3%)	6 (37.5%)	0.544
CRRT, *n* (%)	3 (6.7%)	3 (10.3%)	0 (0.0%)	0.542
Vasopressors use, *n* (%)	4 (8.9%)	4 (13.8%)	0 (0.0%)	0.281
Blood transfusion (7 days), *n* (%)	11 (24.4%)	9 (31.0%)	2 (12.5%)	0.279
Graft dysfunction (7 days), *n* (%)	3 (6.7%)	3 (10.3%)	0 (0.0%)	0.542
Clinical outcomes, median (IQR)				
ICU stay (h)	67.2 (43.5–115.9)	91.0 (47.4–137.2)	53.9 (40.2–67.3)	0.028
Intubation time (h)	18.2 (15.2–43.9)	22.1 (16.0–69.1)	15.8 (13.7–22.0)	0.055
Total hospitalization (days)	33.0 (23.0–54.0)	44.0 (30.0–63.0)	23.0 (21.0–35.2)	0.009
Postoperative stay (days)	27.0 (20.0–37.0)	29.0 (22.0–50.0)	21.0 (18.8–25.5)	0.016

ICU, intensive care unit; rATG, rabbit anti-thymocyte globulin; CRRT, continuous renal replacement therapy.

Among the 29 infected recipients, pulmonary infection was the predominant site (89.7%), followed by abdominal (24.1%) and bloodstream (17.2%) infections. The majority of infected recipients had single-site involvement (75.9%), while 24.1% experienced multi-site infections (**Supplementary Figure S1**).

### Pathogen detection rates and concordance between mNGS and culture

A total of 124 samples were collected from 45 donors across three compartments: sputum (*n* = 44), EDTA blood (*n* = 35), and OPS (*n* = 45). The majority of donors (77.8%) had all three sample types collected concurrently. For recipients (97 samples in total), four types of samples were tested: EDTA blood (*n* = 42), ascites (*n* = 32), sputum (*n* = 20), and OPS (*n* = 2).

At the patient level, mNGS achieved substantially higher overall positivity than conventional culture in both donors (95.6% *vs*. 80.0%, *p* = 0.016) and recipients (64.4% *vs*. 22.2%, *p* < 0.001). This advantage was consistent across all pathogen categories. In donors, fungal detection was markedly higher by mNGS than culture (40.0% *vs*. 15.6%, *p* = 0.003), and viral pathogens were identified exclusively by mNGS (6.7% *vs*. 0%, *p* = 0.250). In recipients, similar patterns were observed: fungal positivity was 26.7% by mNGS *versus* 8.9% by culture (*p* = 0.039), and viruses were detected in 17.8% of the recipients by mNGS alone (*p* = 0.008) (**Figure 1A**). At the sample level, mNGS consistently outperformed culture across all matrices (except for recipient ascites). The highest detection rates were observed in donor sputum (mNGS overall with 93.2% *vs*. culture with 77.3%, *p* = 0.0157) and recipient sputum (mNGS with 85.0% *vs*. culture with 30.0%, *p* = 0.001), followed by donor blood (mNGS overall with 62.9% *vs*. culture with 2.9%, *p* < 0.001) and recipient blood (mNGS with 40.5% *vs*. culture with 0%, *p* < 0.001). With the exception of fungal detection in the donor OPS and recipient ascites, mNGS demonstrated significantly higher bacterial and fungal detection rates than culture across all other sample types from both donors and recipients (*p* < 0.05) (**Figure 1B**).

**Figure 1:**
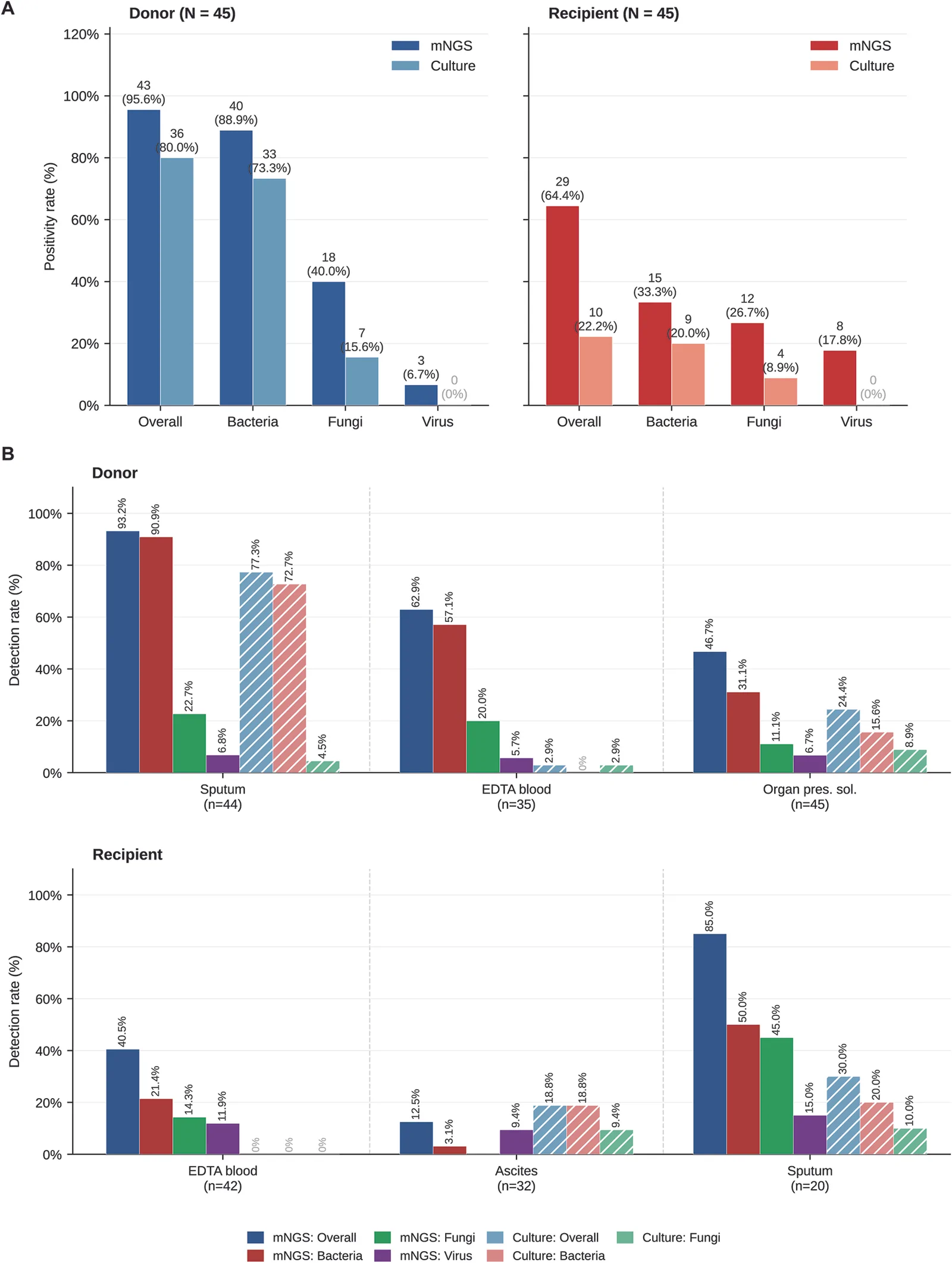
Pathogen detection rates of metagenomic next-generation sequencing (mNGS) *versus* conventional culture in donors and recipients. **(A)** Patient-level overall positivity rates for mNGS and culture in donors (*n* = 45) and recipients (*n* = 45). **(B)** Sample-level detection rates for mNGS and culture in donor sample types and recipient sample types.

Sample-level agreement analysis revealed that, among 124 donor samples, 36.3% (45/124) were positive by both mNGS and culture, 31.5% (39/124) by mNGS alone, and only 0.8% (1/124) by culture alone, with 31.5% (39/124) negative by both. In recipients (97 samples), 9.3% (9/97) were dually positive, 32% (31/97) positive by mNGS alone, 5.2% (5/97) by culture alone, and 54% (52/97) negative by both methods (**Figure 2A**). After accounting for within-subject clustering using GEE, the overall mNGS positivity advantage over culture remained statistically significant in both donors (OR = 3.56, 95%CI = 2.59–4.91, *p* < 0.001) and recipients (OR = 4.20, 95%CI = 2.02–8.75, *p* < 0.001), consistent with the primary unclustered analysis (**Supplementary Table S1**). Among the dually positive samples, pathogen-level concordance was predominantly partial in both donors (75.6%) and recipients (77.8%), with complete concordance achieved in 17.8% and 22.2%, respectively. Complete discordance was observed in 6.7% of donor samples and 0% of recipient samples (**Figure 2B**).

**Figure 2:**
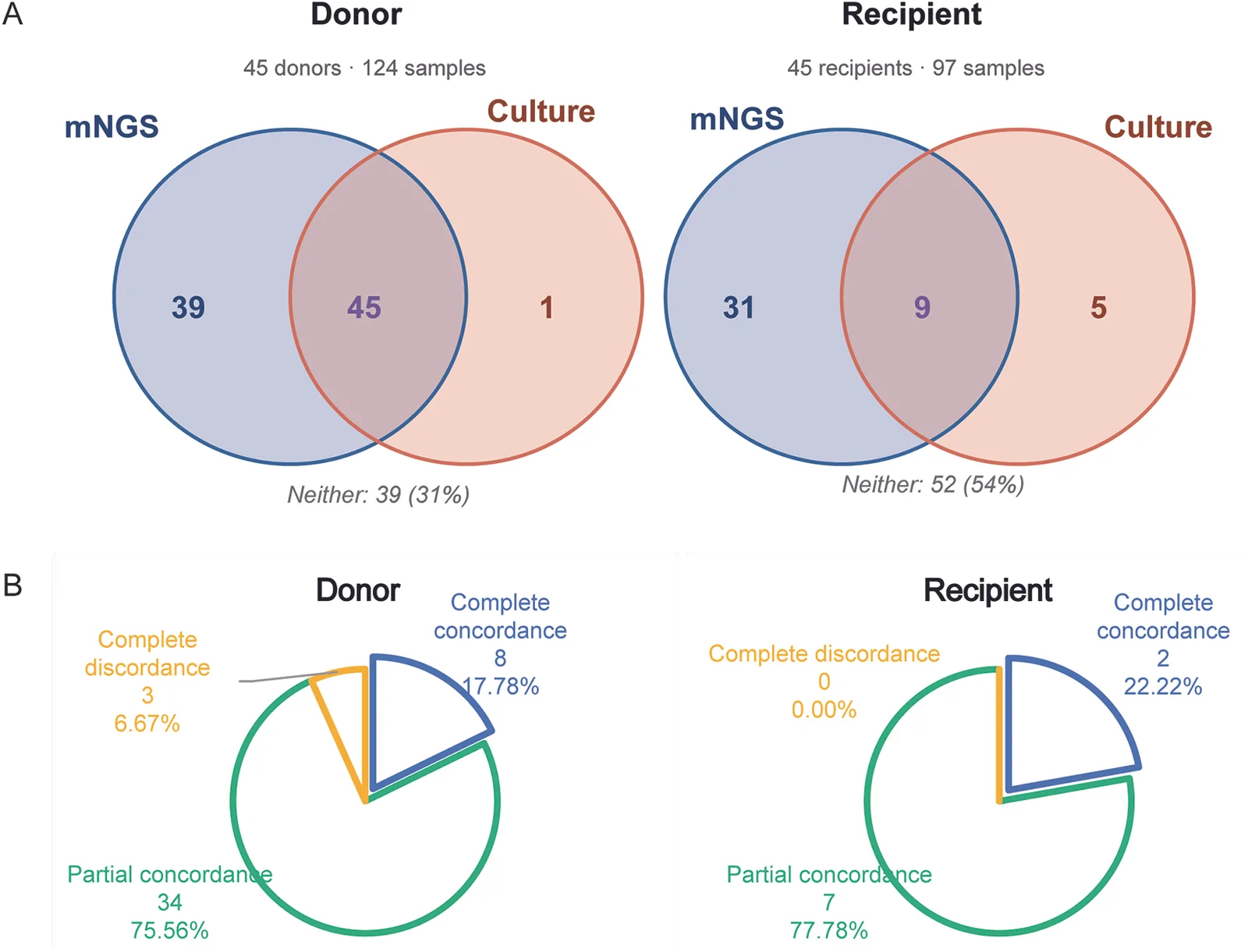
Concordance between metagenomic next-generation sequencing (mNGS) and conventional culture at the sample and pathogen levels in donors and recipients. **(A)** Venn diagram of the sample-level concordance between mNGS and culture for donor samples (*n* = 124) and recipient samples (*n* = 97). **(B)** Pie chart showing the pathogen-level concordance among dually positive donor samples (*n* = 45 samples positive by both methods) and recipient samples (*n* = 9).

For recipient patients, using the clinical adjudicated diagnosis of post-transplant infection as the reference standard, we further analyzed the diagnostic value of mNGS and conventional culture at both the patient level and across different sample types. At the overall patient level, mNGS demonstrated a sensitivity of 69.0% (95%CI = 50.8%–82.7%) and a specificity of 43.8% (95%CI = 23.1%–66.8%), whereas traditional culture showed a sensitivity of 31.0% (95%CI = 17.3%–49.2%) and a specificity of 93.8% (95%CI = 71.7%–98.9%). This trade-off pattern between sensitivity and specificity remained consistent across all sample types. Sputum mNGS exhibited the highest sensitivity (84.6%), but the lowest specificity (14.3%). Blood and ascites mNGS showed moderate sensitivity (44.4% and 14.3%, respectively) with relatively higher specificity. The specificity of traditional culture for both blood and sputum remained consistently at 100% (**Figure 3**). Because the clinically adjudicated reference diagnosis may have been influenced by the mNGS results (i.e., potential incorporation bias), these estimates—in particular the sensitivity of mNGS—should be interpreted with caution.

**Figure 3:**
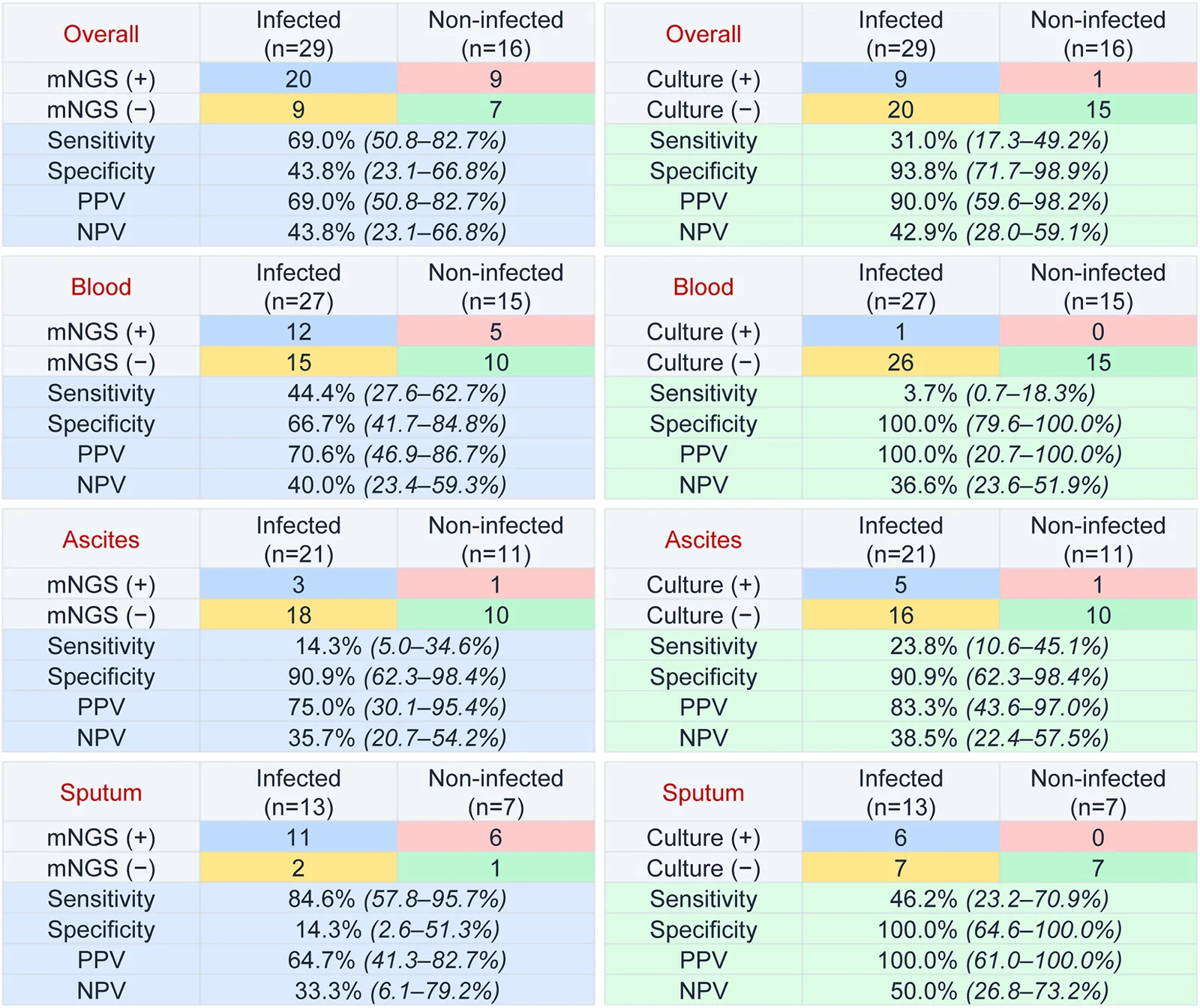
Diagnostic performance of metagenomic next-generation sequencing (mNGS) and conventional culture for post-transplant infection detection in recipients. mNGS, metagenomic next-generation sequencing; PPV, positive predictive value; NPV, negative predictive value; CI, confidence interval.

### Pathogen spectrum identified by mNGS

The pathogen landscape identified across donor and recipient samples was broad and diverse, with mNGS substantially expanding detection beyond conventional culture, particularly for fungi, viruses, and fastidious organisms. In donors, *Corynebacterium striatum* and *S. aureus* were the predominant Gram-positive organisms in the sputum. Gram-negative species were more diverse, with *K. pneumoniae*, *Acinetobacter baumannii*, *Pseudomonas aeruginosa*, and *Stenotrophomonas maltophilia* representing the most frequently detected pathogens. Fungal pathogens were dominated by *Candida* species (*Candida parapsilosis*, *Candida tropicalis*, and *Candida albicans*). Rare molds, including *Rhizomucor pusillus*, *Lichtheimia ramosa*, and *Cunninghamella bertholletiae*, were identified exclusively by mNGS. Viral pathogens, including human parvovirus B19, CMV, human alphaherpesvirus 1, and hepatitis B virus (HBV), were detected solely by mNGS (**Figure 4A**).

**Figure 4:**
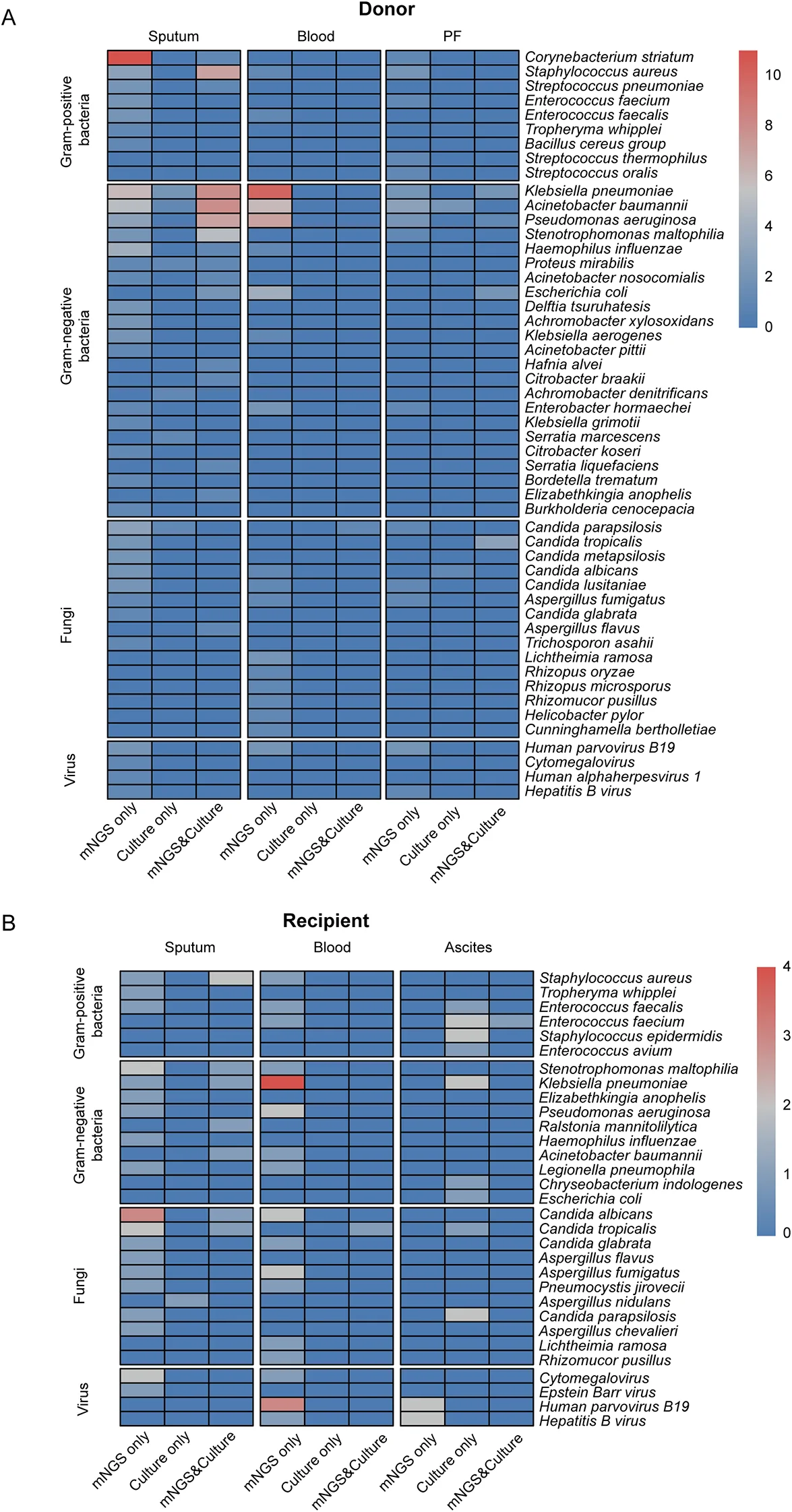
Pathogen spectrum identified by metagenomic next-generation sequencing (mNGS) and conventional culture across donor and recipient sample types. **(A)** Heatmap of the pathogens detected in donor samples [sputum, EDTA blood, and organ preservation fluid (PF)], stratified by detection method [metagenomic next-generation sequencing (mNGS) only, culture only, and both mNGS and culture]. **(B)** Heatmap of the pathogens detected in recipient samples (sputum, blood, and ascites), in the same format.

In recipients, *S. aureus* and *K. pneumoniae* were the leading Gram-positive and Gram-negative pathogens, respectively, with *K. pneumoniae* displaying the highest signal intensity in blood samples. *C. albicans*, *C. tropicalis*, and *Aspergillus* species were the dominant fungi. Critically, *P. jirovecii*—a clinically significant opportunistic pathogen undetectable by conventional culture—was identified exclusively by mNGS. Viral detections included CMV, EBV, human parvovirus B19, and HBV, all via mNGS alone (**Figure 4B**).

### Possible donor-to-recipient pathogen transmission

To investigate possible donor-derived pathogen transmission, we systematically compared the microorganisms identified in matched donor–recipient pairs. There were 11 possible donor-derived transmission events identified across 11 donor–recipient pairs, involving six pathogens, and six of the 11 recipients with possible donor-derived transmission (54.5%) developed clinical infections (**Table 2**). *K. pneumoniae* was the most frequently transmitted organism (five of 11 events). Among the five affected recipients, three developed clinical infections (pulmonary, abdominal, and/or bloodstream). Transmission of *S. aureus* did not cause infection in recipients. Human parvovirus B19 was transmitted in two events, with one recipient developing pulmonary infection. In the HBV-positive pair, the recipient was clinically diagnosed with pulmonary infection after transplantation. However, because HBV was detected in the recipient’s blood and ascites (concordant with the donor’s sputum and OPS) and is not a typical respiratory pathogen, this detection is described as a possible donor-derived viral detection event rather than the causative pathogen of the pulmonary infection. Fungal transmission events involved *C. parapsilosis* (associated with pulmonary and abdominal infection) and *Candida glabrata* (no overt infection).

**Table 2:** Possible donor-to-recipient pathogen transmission events identified by metagenomic next-generation sequencing (mNGS).

Donor–recipient pair	Clinical infection	Transmitted pathogen	Donor detection (sample type, method)	Recipient detection (sample type, method)	Infection site(s) in recipient
3	No	Human parvovirus B19	mNGS: sputum, blood, OPS	mNGS: blood, ascites	–
9	Yes	Human parvovirus B19	mNGS: sputum, blood, OPS	mNGS: blood, ascites	Pulmonary
10	Yes	*Klebsiella pneumoniae*	mNGS + culture: OPS mNGS: blood	mNGS: blood culture: ascites	Pulmonary, abdominal, bloodstream
13	No	*Klebsiella pneumoniae*	mNGS + culture: sputum mNGS: blood	mNGS: blood	–
15	No	*Staphylococcus aureus*	mNGS: sputum	mNGS: sputum	–
17	No	*Candida glabrata*	mNGS: sputum	mNGS: sputum	–
18	Yes	*Klebsiella pneumoniae*	mNGS: blood culture: OPS	mNGS + culture: BALF	Pulmonary, bloodstream
19	Yes	Hepatitis B virus	mNGS: sputum, OPS	mNGS: blood, ascites	Pulmonary
29	No	*Klebsiella pneumoniae*	mNGS: sputum	mNGS: blood, sputum	–
32	Yes	*Candida parapsilosis*	mNGS: OPS	culture: ascites	Pulmonary, abdominal
37	Yes	*Klebsiella pneumoniae*	mNGS + culture: sputum mNGS: blood	mNGS + culture: sputum mNGS: blood	Pulmonary

*mNGS*, metagenomic next-generation sequencing; *OPS*, organ preservation solution; *BALF*, bronchoalveolar lavage fluid.

### Impact on antimicrobial treatment decisions for receptors

The mNGS findings prompted antimicrobial treatment adjustment in 24 of 45 recipients (53.3%), including 20 infected (69.0%) and four non-infected (25.0%) recipients. In contrast, the culture results alone did not inform treatment modifications in any recipients beyond those who were also positive by mNGS. Among the mNGS-informed modifications in infected recipients, escalation (40%), addition of anti-Gram-positive agents (35%), combination therapy (35%), and antifungal modification (25%) were the most frequent intervention categories. Antiviral additions (10%) and de-escalation (5%) were exclusively mNGS-driven, reflecting the broader pathogen spectrum captured by this technology (**Figure 5**).

**Figure 5:**
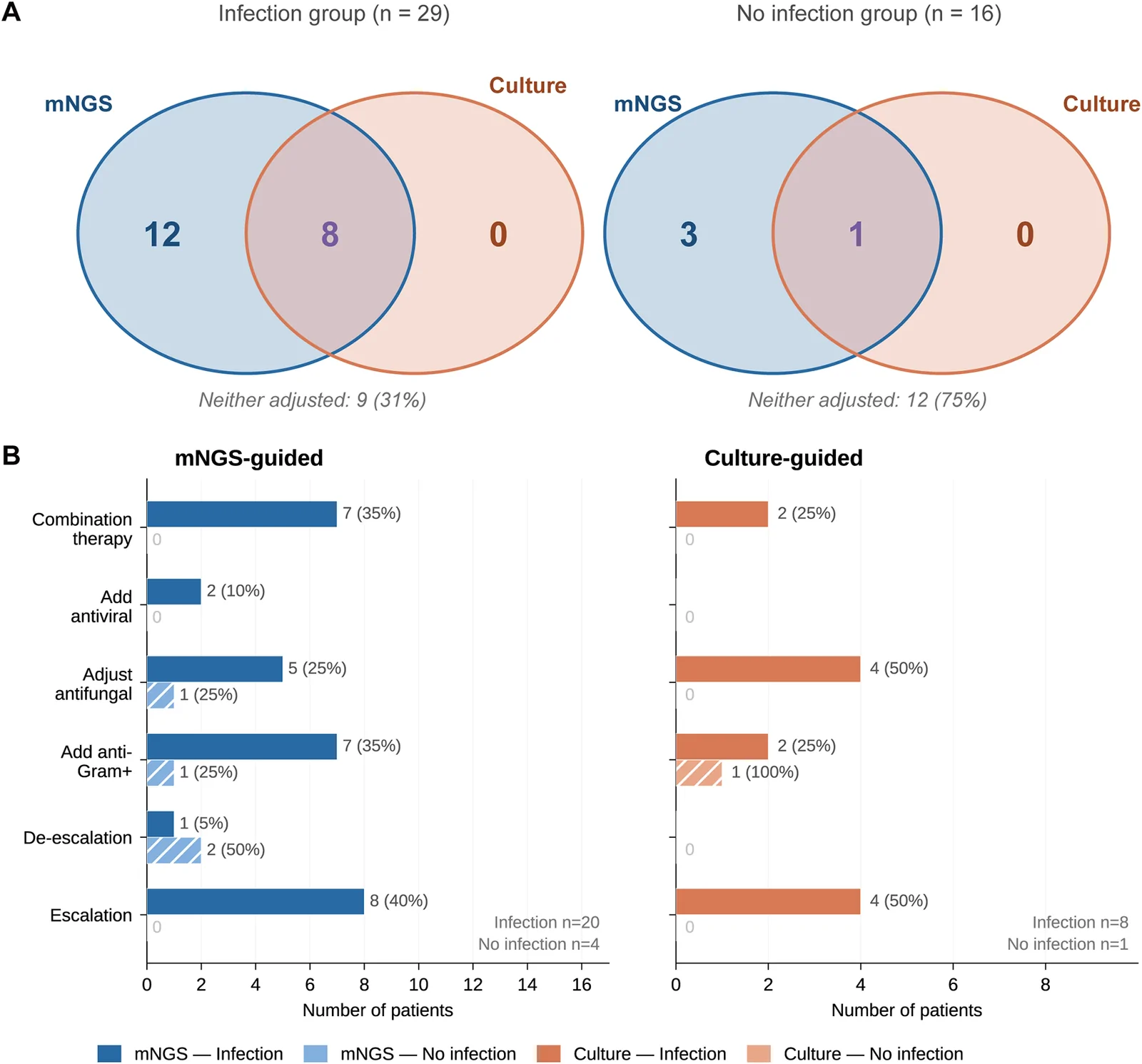
Antimicrobial treatment modifications informed by metagenomic next-generation sequencing (mNGS) and conventional culture in recipients. **(A)** Number and proportion of recipients with treatment adjustment informed by the mNGS and culture results, stratified by infection status (infected, *n* = 29; non-infected, *n* = 16). **(B)** Types of antimicrobial treatment adjustments in the mNGS-informed cohort (infected, *n* = 20; non-infected, *n* = 4) and the culture-informed cohort (infected, *n* = 8; non-infected, *n* = 1).

Among the nine recipients whose treatment was adjusted based on both methods concurrently, the Jaccard similarity index between the mNGS- and culture-informed modification categories was 1.00 (complete concordance) in six cases and 0.50–0.67 (partial concordance) in three cases, with no complete discordance. In all three partial-concordance cases, mNGS identified additional adjustment categories beyond those prompted by culture (**Table 3**).

**Table 3:** Concordance of antimicrobial treatment adjustment categories between metagenomic next-generation sequencing (mNGS)-informed and culture-informed decisions in dually modified recipients.

Recipient ID	Clinical infection	mNGS-informed modification(s)	Culture-informed modification(s)	Shared categories	Jaccard index
10	Yes	Escalation Adjust antifungal	Escalation Adjust antifungal	Escalation Adjust antifungal	1.00
16	Yes	Add anti-Gram+ Combination therapy	Combination therapy	Combination therapy	0.50
18	Yes	Escalation	Escalation	Escalation	1.00
20	Yes	Adjust antifungal	Adjust antifungal	Adjust antifungal	1.00
26	Yes	Add anti-Gram+ Add antiviral Escalation	Add anti-Gram+ Escalation	Add anti-Gram+ Escalation	0.67
34	No	Add anti-Gram+	Add anti-Gram+	Add anti-Gram+	1.00
36	Yes	Add antiviral Adjust antifungal Combination therapy Escalation	Adjust antifungal Combination therapy	Adjust antifungal Combination therapy	0.50
37	Yes	Adjust antifungal Escalation	Adjust antifungal Escalation	Adjust antifungal Escalation	1.00
40	Yes	Add anti-Gram+	Add anti-Gram+	Add anti-Gram+	1.00

Jaccard index = number of shared categories/number of unique categories across both methods. A value of 1.00 indicates complete concordance, while 0 indicates complete discordance.

Recipients who underwent mNGS-informed treatment modifications had significantly higher post-transplant infection rates compared with those who did not (83.3% *vs*. 42.9%, Fisher’s exact *p* = 0.006), indicating that mNGS-informed modification was concentrated in the highest-risk subgroup.

### Risk factors for post-transplant infection

In the univariate logistic regression, liver cancer showed an exploratory association with lower post-transplant infection risk (OR = 0.10, 95%CI = 0.02–0.46, *p* = 0.003), consistent with the analysis of the baseline characteristics. ICU duration per 10-h increment showed a borderline association (OR = 1.11, 95%CI = 0.99–1.24, *p* = 0.088). No other clinical, perioperative, or donor-derived microbiological variable reached statistical significance at the univariate level, although donor mNGS fungal detection (OR = 2.80, 95%CI = 0.73–10.75, *p* = 0.134) and blood transfusion within 7 days (OR = 3.15, 95%CI = 0.59–16.86, p = 0.180) showed trends toward significance (**Figure 6**).

**Figure 6:**
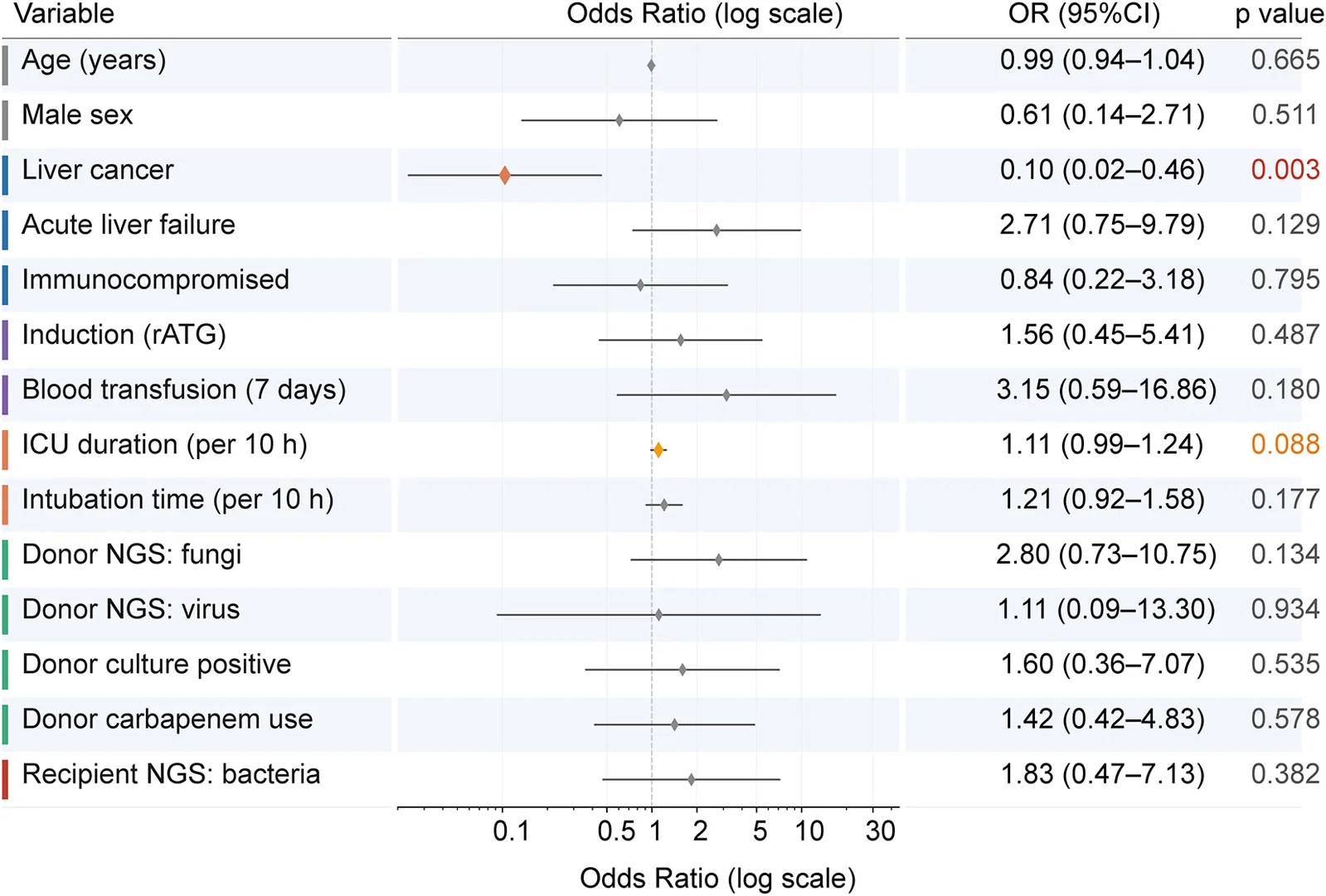
Univariate logistic regression analysis of the risk factors for post-transplant infection. Forest plot displaying odds ratios (ORs) and 95% confidence intervals (CIs) from the univariate logistic regression analysis of the clinical, perioperative, microbiological, and donor-derived variables as predictors of post-transplant infection in recipients (*n* = 45). Variables are color-coded by category: recipient characteristics (*gray*), underlying disease (*blue*), perioperative variables (*purple*), clinical outcomes (*yellow*), donor-derived pathogen findings (*green*), and recipient metagenomic next-generation sequencing (mNGS) results (*red*). Variables with complete separation (CRRT, vasopressor use) were excluded from the analysis. The *dashed vertical line* at OR = 1 represents no effect. *Orange diamonds* indicate *p* < 0.05, while *yellow diamonds* indicate *p* < 0.10. *HCC*, hepatocellular carcinoma; *rATG*, rabbit anti-thymocyte globulin; *ICU*, intensive care unit; *CRRT*, continuous renal replacement therapy.

## Discussion

In this matched-pair cohort study of 45 LT donor–recipient pairs, it was demonstrated that mNGS substantially outperformed conventional culture in pathogen detection across multiple biological compartments in both donors and recipients, enabled the identification of possible donor-derived transmission events involving clinically important pathogens, and drove the majority of antimicrobial treatment adjustments in infected recipients. Taken together, these findings suggest that the addition of mNGS to conventional culture may enhance peri-transplant microbiological surveillance and antimicrobial stewardship. However, given the exploratory, single-center nature of this study, prospective multicenter validation is required before routine implementation can be recommended.

The overall mNGS positivity rates of 95.6% in donors and 64.4% in recipients substantially exceeded those of conventional culture (80.0% and 22.2%, respectively), consistent with findings from prior studies of mNGS in post-LT infection. Huang et al. (2022) reported that donor mNGS positivity (64.3%) significantly exceeded that of traditional methods (28.6%, *p* < 0.05), and Zhuang et al. (2024) demonstrated high mNGS predictive performance for post-LT pulmonary infections with superior sensitivity compared with culture (Huang et al., 2022; Zhuang et al., 2024). Our data extend these observations by providing a systematic multi-compartment comparison. The advantage of mNGS was most pronounced for fungal pathogens—detected at 40.0% *versus* 15.6% in donors—and was absolute for viral pathogens, which were identified in 6.7% of donors and 17.8% of recipients exclusively by mNGS. This finding aligns with the well-established inability of routine culture to detect viruses and the recognized diagnostic superiority of molecular methods for organisms such as CMV, EBV, and *P. jirovecii* (Zheng et al., 2025).

Importantly, our concordance analysis revealed that mNGS and culture are best understood as complementary rather than interchangeable diagnostic modalities. While 36% of the donor samples and 9% of the recipient samples were positive by both methods, a further 31% of donor and 32% of recipient samples were detected solely by mNGS, with culture alone identifying 1% and 5%, respectively. At the pathogen level, complete concordance was achieved in only 18%–22% of dually positive samples, with partial concordance—meaning overlapping but non-identical pathogen profiles—predominating in 75%–78%. This pattern of partial concordance implies that each method consistently reveals a distinct subset of the pathogen burden not captured by the other, underscoring the rationale for an integrated dual-method strategy. A comparable complementarity profile has been reported across other mNGS studies in immunocompromised cohorts, where mNGS and culture together detected substantially more organisms than either method alone (Huang et al., 2022; Wang et al., 2025).

The detection of *P. jirovecii* exclusively by mNGS in our recipient cohort is of particular clinical importance. *P. jirovecii* pneumonia (PJP) is a severe opportunistic infection in SOT recipients that carries significant mortality if not promptly treated. However, conventional diagnosis relies on special staining methods and biomarker assays with suboptimal performance (Huang et al., 2024). Prior studies in kidney transplant recipients have demonstrated mNGS sensitivity rates of 83%–100% for PJP, substantially exceeding those of conventional approaches (Wang et al., 2025). Similarly, the identification of rare invasive molds—including *C. bertholletiae*, *R. pusillus*, and *L. ramosa*—exclusively through mNGS in our cohort highlights the unique ability of this technology to detect clinically dangerous but culture-resistant organisms that may otherwise go unidentified until clinical deterioration is advanced (Pappas et al., 2010).

A clinically consequential finding of this study was the identification of 11 possible donor-to-recipient pathogen transmission events across 11 pairs, involving six distinct organisms, with 54.5% of affected recipients developing clinical infections. *K. pneumoniae* was the most frequently transmitted pathogen, responsible for five events—a finding consistent with the established epidemiology of multidrug-resistant (MDR) Gram-negative transmission in SOT. This mirrors broader epidemiological patterns in LT infections: a recent 13-year single-center cohort similarly identified *Klebsiella* spp. as the leading causative organism of post-transplant infection, with carbapenem resistance detected in nearly two-thirds of isolates and carbapenem-resistant Gram-negative organisms accounting for the majority of infection-related deaths (Kurt et al., 2026). Prior reports have documented fatal donor-derived carbapenem-resistant *K. pneumoniae* infections in liver and kidney transplant recipients, with transmission occurring via the allograft or OPS (Giani et al., 2014; Varotti et al., 2016). The fact that three of five recipients with donor-derived *K. pneumoniae* developed multi-site clinical infections, including bloodstream and abdominal involvement, highlights the virulence of this organism in the profoundly immunocompromised post-transplant setting. A recent study of DDIs in a large Chinese SOT cohort reported incidence rates of 16.6%–19.4% in kidney and liver recipients, with MDR organisms prevalent among deceased donors and preservation fluid contamination as a primary transmission route. The integration of mNGS into donor screening was identified as a key strategy for enabling timely intervention (Zhou et al., 2026). Our data also demonstrated possible donor-derived transmission of HBV and human parvovirus B19, both identified exclusively by mNGS and absent from culture-based screening. These viral transmissions are expected in the context of immunosuppressed recipients unable to mount effective antiviral responses, and their detection has direct implications for prophylaxis and surveillance protocols (Nam et al., 2018; Grossi et al., 2024). Notably, the identification of these organisms in OPS—a sample type not routinely subjected to microbiological testing in many transplant centers—further underscores the value of comprehensive multi-compartment donor mNGS profiling. Current guidelines advocate for systematic culture of preservation fluid for fungal contamination in some European centers; however, comprehensive viral and bacterial profiling via mNGS is not yet standard practice (Grossi et al., 2024).

Our diagnostic performance analysis revealed a characteristic trade-off between mNGS (higher sensitivity, but lower specificity) and culture (lower sensitivity, but higher specificity) that was consistent across sample types. At the overall patient level, the mNGS sensitivity of 69.0% far exceeded the culture sensitivity of 31.0%, while the culture specificity of 93.8% substantially exceeded that of mNGS (43.8%). This pattern mirrors findings reported in other transplant and immunocompromised settings, where mNGS sensitivity values of 58%–85% and culture sensitivity values of 17%–46% have been documented (Lin et al., 2023; Zhuang et al., 2024; Zheng et al., 2025). The lower specificity of mNGS likely reflects detection of colonizing organisms, environmental contaminants, or subclinical microbial signals that are detectable but not responsible for active infection. Consequently, pathogen-level detection may not correspond directly to patient-level infection, even when the detected organism is considered clinically relevant. This distinction is particularly important when interpreting positive mNGS results. In addition, because clinicians were not blinded to the mNGS results in our retrospective study, incorporation bias cannot be excluded and may have influenced the estimated diagnostic performance. Therefore, mNGS should not be used as a stand-alone screening tool, but rather interpreted in conjunction with clinical, radiological, and other microbiological evidence.

Sputum emerged as the highest-yield sample type in our cohort, with mNGS sensitivity of 84.6% and conventional culture specificity of 100%, the latter ensuring that any positive sputum culture carries strong confirmatory value. This finding is congruent with the predominance of pulmonary infection (89.7%) in our infected recipients and with published data showing that BALF/sputum mNGS achieves higher positivity rates than blood-based matrices (Lin et al., 2023; Wu et al., 2024). Conventional blood culture demonstrated the lowest sensitivity (3.7%) with perfect specificity (100%), consistent with the known poor yield of blood cultures in this setting and confirming that positive blood cultures represent highly reliable diagnostic events that warrant immediate clinical action. The low sensitivity of both methods for ascites detection (14.3%–23.8%) suggests that abdominal infection surveillance remains particularly challenging and that neither mNGS nor culture alone is sufficient to exclude peritoneal infection in clinically suspicious cases.

A central contribution of this study is the quantification of the impact of mNGS on antimicrobial treatment decisions. The mNGS findings were temporally associated with treatment modifications in 69.0% of infected recipients. In contrast, the culture results alone informed no additional modifications beyond those already associated with the mNGS findings. The spectrum of mNGS-informed modifications was notably broader than that achievable through culture, including antiviral additions (10%) and de-escalation strategies (5%), categories that culture cannot support given its inability to detect viral pathogens and its characteristic diagnostic delay. This observation is consistent with broader evidence that mNGS-informed therapy in immunocompromised patients is associated with treatment transitions, combination therapy, and antimicrobial downgrading that would not otherwise be initiated (Peng et al., 2024; Zhuang et al., 2024). Of note is that these modifications reflect decision impact rather than proven clinical benefit, and their associations with clinical outcomes merit further investigation.

The high concordance between mNGS- and culture-informed adjustment categories (Jaccard index = 1.00 in six out of nine dual-adjustment cases and 0.50–0.67 in the remaining three) indicates that the mNGS-informed decisions were not discordant with the culture-confirmed findings, but rather extended them by adding additional actionable information—particularly with regard to viral and fungal pathogens. No complete discordance was observed in recipients. Consistent with the interpretation above, these observations support decision impact rather than independent proof of appropriateness or clinical benefit.

The association between liver cancer and lower post-transplant infection risk (univariate OR = 0.10, *p* = 0.003) should be interpreted with caution. This finding was derived from univariate analysis in a small cohort (*n* = 45) and may reflect confounding by underlying disease severity or baseline clinical status: recipients transplanted for hepatocellular carcinoma (HCC) are more likely to have compensated liver function, better nutritional status, and elective rather than emergency transplantation compared with those transplanted for acute liver failure or decompensated cirrhosis. We therefore regard this as an exploratory association rather than as evidence of an independent protective effect of liver cancer.

Several limitations of this study warrant consideration. First, the cohort size of 45 donor–recipient pairs limits statistical power for multivariable regression and subgroup analyses, and the numerically elevated ORs observed for donor mNGS fungal detection and perioperative blood transfusion may represent clinically meaningful associations that were underpowered to reach significance. Second, our study design did not include whole-genome sequencing or molecular typing to formally confirm clonal identity between the donor and recipient isolates in presumed transmission events. Pathogen transmission was inferred based on species-level concordance in matched pairs, which may have overestimated true transmission; therefore, all transmission events are classified as possible rather than confirmed. Third, the reference standard for post-transplant infection was the clinically adjudicated diagnosis rather than a predefined microbiological or pathological criterion, which may have introduced variability related to clinical judgment. Fourth, because mNGS was performed as part of routine clinical care in this retrospective cohort, the clinicians establishing the final diagnoses were not blinded to the mNGS results. This potential incorporation bias may have inflated the diagnostic performance estimates, in particular the sensitivity, and the estimates should therefore be interpreted with caution. Fifth, although the principal sample-level positivity comparisons accounted for within-subject correlations using GEE, the descriptive sample-level concordance analyses (Cohen’s *κ* and the Jaccard index) treated each sample as an independent observation. Given repeated sampling from the same donors and recipients, these descriptive indices may not fully account for within-subject clustering. Sixth, although a structured multidisciplinary adjudication process was used to distinguish colonization from infection, this determination—particularly for organisms with high colonization potential such as *S. aureus* and *C. striatum*—inevitably retains an element of clinical judgment, and inter-observer variability cannot be fully excluded. Finally, the single-center design and patient population in China may have limited generalizability to settings with different donor pool characteristics and local epidemiology. Prospective multicenter studies with genomic confirmation of transmission and standardized mNGS interpretation frameworks are needed to validate and extend these findings.

## Conclusions

This retrospective matched-pair study suggests that mNGS substantially expands pathogen detection beyond conventional culture across donor and recipient samples in LT and allows for the systematic identification of clinically relevant donor-derived pathogen transmission—particularly for *K. pneumoniae* and opportunistic viruses. Moreover, treatment modifications were temporally associated with the mNGS results in the large majority of infected recipients. The complementary performance profiles of mNGS and culture support an integrated dual-method diagnostic approach. These findings suggest a potential role for mNGS as an adjunctive tool for peri-transplant microbiological surveillance and antimicrobial decision-making. However, prospective multicenter studies with standardized interpretation frameworks and molecular confirmation are required before routine integration into clinical protocols can be recommended.

## Data Availability

The data that support the findings of this study are available from the corresponding author upon reasonable request.

## References

[B1] Giani T. Conte V. Mandalà S. D'Andrea M. M. Luzzaro F. Conaldi P. G. . (2014). Cross-infection of solid organ transplant recipients by a multidrug-resistant Klebsiella pneumoniae isolate producing the Oxa-48 carbapenemase, likely derived from a multiorgan donor. J. Clin. Microbiol. 52, 2702–2705. doi: 10.1128/jcm.00511-14 24759725 PMC4097706

[B2] Grossi P. A. Wolfe C. Peghin M. (2024). Non-standard risk donors and risk of donor-derived infections: From evaluation to therapeutic management. Transpl Int. 37, 12803. doi: 10.3389/ti.2024.12803 39416809 PMC11479921

[B3] Huang Z. Hu B. Li J. Feng M. Wang Z. Huang F. . (2024). Metagenomic versus targeted next-generation sequencing for detection of microorganisms in bronchoalveolar lavage fluid among renal transplantation recipients. Front. Immunol. 15, 1443057. doi: 10.3389/fimmu.2024.1443057 39253087 PMC11381253

[B4] Huang J. F. Miao Q. Cheng J. W. Huang A. Guo D. Z. Wang T. . (2022). Metagenomic next-generation sequencing versus traditional laboratory methods for the diagnosis and treatment of infection in liver transplantation. Front. Cell. Infect. Microbiol. 12, 886359. doi: 10.3389/fcimb.2022.886359 35782119 PMC9245428

[B5] Jiang Z. Gai W. Zhang X. Zheng Y. Jin X. Han Z. . (2024). Clinical performance of metagenomic next-generation sequencing for diagnosis of pulmonary Aspergillus infection and colonization. Front. Cell. Infect. Microbiol. 14, 1345706. doi: 10.3389/fcimb.2024.1345706 38606292 PMC11007027

[B6] Kurt E. K. Bulut R. Kandemir B. Keskin P. B. Şentürk M. Bıyık M. . (2026). Evaluation of infections developing in liver transplant patients: Thirteen-year experience of a single center. Transplant. Proc. 58, 760–768. doi: 10.1016/j.transproceed.2026.03.013 41997770

[B7] Lin T. Tu X. Zhao J. Huang L. Dai X. Chen X. . (2023). Microbiological diagnostic performance of metagenomic next-generation sequencing compared with conventional culture for patients with community-acquired pneumonia. Front. Cell. Infect. Microbiol. 13, 1136588. doi: 10.3389/fcimb.2023.1136588 37009509 PMC10061305

[B8] Liu Y. Zhang R. Yao B. Yang J. Ge H. Zheng S. . (2022). Metagenomics next-generation sequencing provides insights into the causative pathogens from critically ill patients with pneumonia and improves treatment strategies. Front. Cell. Infect. Microbiol. 12, 1094518. doi: 10.3389/fcimb.2022.1094518 36710980 PMC9880068

[B9] Meng F. Zhu C. Zhu C. Sun J. Chen D. Ding R. . (2024). Epidemiology and pathogen characteristics of infections following solid organ transplantation. J. Appl. Microbiol. 135, lxae292. doi: 10.1093/jambio/lxae292 39567858

[B10] Mezochow A. K. Schaubel D. E. Peyster E. G. Lewis J. D. Goldberg D. S. Bittermann T. (2024). Hospitalizations for opportunistic infections following transplantation and associated risk factors: A national cohort study of Medicare beneficiaries. Transpl Infect. Dis. 26, e14317. doi: 10.1111/tid.14317 38852064 PMC11315637

[B11] Miller S. Naccache S. N. Samayoa E. Messacar K. Arevalo S. Federman S. . (2019). Laboratory validation of a clinical metagenomic sequencing assay for pathogen detection in cerebrospinal fluid. Genome Res. 29, 831–842. doi: 10.1101/gr.238170.118 30992304 PMC6499319

[B12] Nam H. Nilles K. M. Levitsky J. Ison M. G. (2018). Donor-derived viral infections in liver *transplantation*. Transplantation 102, 1824–1836. doi: 10.1097/tp.0000000000002326 29979345 PMC8431779

[B13] Pappas P. G. Alexander B. D. Andes D. R. Hadley S. Kauffman C. A. Freifeld A. . (2010). Invasive fungal infections among organ transplant recipients: Results of the Transplant-Associated Infection Surveillance Network (Transnet). Clin. Infect. Dis. 50, 1101–1111. doi: 10.1086/651262 20218876

[B14] Peng H.-B. Liu Y. Hou F. Zhao S. Zhang Y.-Z. He Z.-Y. . (2024). Clinical application of metagenomic next-generation sequencing (Mngs) in patients with early pulmonary infections after liver transplantation. Infect. Drug Resist. 17, 5685–5698. doi: 10.2147/IDR.S483684 39717063 PMC11665138

[B15] Van Den Daele C. Martiny D. Etienne I. Kemlin D. Roussoulières A. Sokolow Y. . (2024). Monocentric, retrospective study on infectious complications within one year after solid-organ transplantation at a Belgian university hospital. Microorganisms 12, 755. doi: 10.3390/microorganisms12040755 38674699 PMC11052249

[B16] Varotti G. Dodi F. Marchese A. Terulla A. Bertocchi M. Fontana I. (2016). Fatal donor-derived carbapenem-resistant Klebsiella pneumoniae infection in a combined kidney-pancreas transplantation. Case Rep. Transplant. 2016, 7920951. doi: 10.1155/2016/7920951 27822401 PMC5086363

[B17] Wang Q. Ding H. Hao Z. Liao G. (2025). Metagenomic next-generation sequencing enhances diagnosis of fungal infections in kidney transplant recipients: A retrospective study. Front. Cell. Infect. Microbiol. 15, 1667475. doi: 10.3389/fcimb.2025.1667475 41668736 PMC12883835

[B18] Wu H. Cao H. Gao X. Shi C. Wang L. Gao B. (2024). The role of metagenomic next-generation sequencing in diagnosing and managing post-kidney transplantation infections. Front. Cell. Infect. Microbiol. 14, 1473068. doi: 10.3389/fcimb.2024.1473068 39839264 PMC11747774

[B19] Yang H. Xu N. Yan M. Yang L. Wen S. Wang S. . (2024). Comparison of metagenomic next-generation sequencing and conventional culture for the diagnostic performance in febrile patients with suspected infections. BMC Infect. Dis. 24, 350. doi: 10.1186/s12879-024-09236-w 38532348 PMC10964532

[B20] Zhao D. Guo L. Lian D. Gu Y. Yan X. Hu H. . (2022). Diagnostic value and clinical application of Mngs for post-liver transplantation infection: A cross-sectional study with case reports. Front. Microbiol. 13, 919363. doi: 10.3389/fmicb.2022.919363 35847093 PMC9283086

[B21] Zheng X. Zou W. Zou S. Ye J. Bao Z. Song Y. (2025). Diagnostic significance of metagenomic next-generation sequencing in immunocompromised patients with suspected pulmonary infection. Immunology 175, 112–122. doi: 10.1111/imm.13911 39988326 PMC11982602

[B22] Zhou Y. M. Cui X. Q. Zhao P. Peng Z. G. Guo N. Sun H. B. . (2026). The species, distribution, resistance of donor-derived pathogens and their impact on solid organ transplant recipients. Front. Immunol. 17, 1777244. doi: 10.3389/fimmu.2026.1777244 41869352 PMC13002418

[B23] Zhuang L. Zhu C. Ma J. Zhu D. Zhu H. Zhong S. . (2024). Predictive performance of metagenomic next generation sequencing in early detection of post-liver transplantation infections. Heliyon 10, e36405. doi: 10.1016/j.heliyon.2024.e36405 39253237 PMC11381781

