# Supplementary material for: Metagenomic next-generation sequencing of matched donor–recipient pairs reveals pathogen transmission dynamics and informs antimicrobial therapy in liver transplantation: a retrospective cohort study
Source: Front Cell Infect Microbiol. 2026 Oct 19;16:1919428. doi: 10.3389/fcimb.2026.1919428 (PMC13724449; doi:10.3389/fcimb.2026.1919428)
Supplement: Supplementary file 1 [file Table1.docx]

Supplementary Material

**Supplementary Table 1**. Sensitivity analysis accounting for within-subject clustering of repeated samples (GEE).

| Group | Samples, n (subjects, n) | mNGS positive, n (%) | Culture positive, n (%) | Naive (unclustered) χ² P | GEE-adjusted OR (95% CI) | GEE P |
| --- | --- | --- | --- | --- | --- | --- |
| Donor | 124 (45) | 84 (67.7%) | 46 (37.1%) | <0.001 | 3.56 (2.59–4.91) | <0.001 |
| Recipient | 97 (45) | 40 (41.2%) | 14 (14.4%) | <0.001 | 4.20 (2.02–8.75) | <0.001 |

GEE, generalized estimating equations; OR, odds ratio (mNGS vs. culture, positive vs. negative), estimated from a GEE binomial model with an exchangeable working correlation structure and robust (sandwich) variance estimator, clustering on subject identity to account for multiple samples (up to 3 per donor; up to 4 per recipient) contributed by the same individual; CI, confidence interval.

**Supplementary Figure 1.** Distribution of infection sites among post-transplant recipients. (A) Proportion of infected recipients (n = 29) with involvement of each anatomical site. (B) Proportion of infected recipients with single-site versus multi-site infection.
